# In the parents’ view: weight perception accuracy, disturbed eating patterns and mental health problems among young adolescents

**DOI:** 10.1186/2050-2974-2-9

**Published:** 2014-03-19

**Authors:** Liv Sand, Bryan Lask, Mari Hysing, Kjell Morten Stormark

**Affiliations:** 1 Regional Centre for Child and Youth Mental Health and Welfare, Uni Health, PO Box 7810, N-5020 Bergen, Norway; 2 Gt Ormond St Hospital for Children NHS Foundation Trust, Great Ormond Street, London WC1N 3JH, UK

**Keywords:** Weight perception, Parent reports, Adolescence, Eating patterns, Mental health

## Abstract

**Background:**

An accurate weight perception has been associated with motivation to change eating habits in the case of under- or overweight. However, recent studies have reported frequent misperceptions among parents and their offspring, both in the form of under- and overestimation of weight status. The aim of the present study was to investigate weight perception accuracy among parents of young adolescents in relation to reports on disturbed eating patterns and mental health problems.

**Methods:**

Weight perception accuracy was assessed among parents of young adolescents (N = 5,781, aged 11 - 13 years) who participated in the ongoing Bergen Child Study (BCS). Parental weight perception was classified in overestimation, underestimation and accurate. Other measures included demographic variables, the parents’ evaluations of disturbed eating patterns and mental health problems among their offspring as well as the adolescents’ own weight perception accuracy.

**Results:**

The parents accurately perceived more than 80% of normal weight adolescents, but nearly 60% of the underweight adolescents were overestimated, and a substantial proportion of overweight girls (34. 8%) and boys (12.8%) were underestimated. In general, parents who were aware of deviations from average weight in their child also reported higher levels of disturbed eating patterns, emotional problems, and behavioral problems. After controlling for demographic factors, the risk of parental over- and underestimation was significantly predicted by weight status, the adolescents’ weight perception accuracy as well as disturbed eating patterns reported by the parents (p < .05).

**Conclusions:**

Adolescents with under- or overweight proved most likely to be misperceived by their parents in this study. The pattern of perceptually correcting deviations from normal weight was interpreted as a positivity bias among the parents. These results suggest that weight perception accuracy should be targeted in family-focused interventions in order to strengthen adequate weight control among young adolescents.

## Background

Weight control in children and adolescents has become an important challenge to public health. Despite efforts to fight the obesity epidemic, a substantial proportion of overweight adolescents are not engaging in appropriate weight reducing behaviors [1], thereby increasing the risk for prolonged obesity and poor emotional well-being into adulthood [2]. In contrast, a growing number of non-overweight adolescents make efforts to reduce their weight by unsupervised diets [3] that may develop into more serious eating pathology. Weight perception has been proposed as an important factor for explaining the relationship between weight status and weight control behavior [4]. An inaccurate weight perception can take the form of both over- and underestimation of personal weight status. Both misperceptions can challenge healthy weight maintenance, as overestimation has been associated with unwarranted and unsafe dieting among adolescents [5], while underestimation among overweight or obese individuals may result in low motivation to reduce excessive weight [6].

Parental attitude and behavior can influence the formation of body image and eating behavior among children and adolescents [7], possibly by modelling effects [8], particularly around meals and initiatives to physical exercise or sedentary activities [9]. Parents’ ability to take action with regard to weight problems is again dependent upon being aware of the weight status of their children [10]. An important basis for this awareness is that parents recognize weight deviations in their offspring [11], and addressing parental weight perception seems important in preventing under- and overweight among children and adolescents.

Earlier research on the accuracy of parent’s weight perceptions has focused mainly on their recognition of *overweight* in children. A review concludes that more than 60% of children with overweight were perceived by their parents of being of normal weight [12]. Comparable results have been reported for samples including both children and adolescents with overweight or obesity [13], although it has been suggested that the accuracy of parental weight perception increases with the age of the offspring [14]. There are mixed findings with regard to the offspring’s gender, as some studies show that overweight boys are more likely to be underestimated than girls [15], while others show either the opposite [16] or no gender effect [17]. The likelihood of parents’ underestimation seem to increase with higher Body Mass Index (BMI) of the offspring [16], and this may increase the risk of maintaining unhealthy eating patterns for children and youth who are already at the upper levels of the weight distribution.

Some studies have also investigated how accurate parents are in estimating the BMI of children who are *under*- or of *normal* weight. While parental estimates of children with normal weight were accurate in the vast majority of the cases, nearly half of the underweight children were overestimated [18]. The high frequency of overestimation for underweight children has been replicated by other studies with Swedish [19] and Norwegian [20] samples. Although there are fewer studies on this specific misperception, the high prevalence found so far and possible risks of not recognizing underweight with regard to development and growth [21] make this an important research topic in addition to the underestimation of excessive weight.

Thus, previous research has shown substantial discrepancies between actual and perceived weight reported by parents that parallel the misperceptions among children and adolescents themselves [22]. Parents generally seem to be least accurate for children who differ most from average weight, as they tend to overestimate if the child is underweight and underestimate if the child is overweight [20]. This implies that parents tend to correct from deviations from average weight, and Akerman and colleagues [19] have proposed that this skewedness can be understood as a *positivity bias.* The general function of this bias is to protect and maintain a positive image of oneself and close ones [23]. From this perspective, under- and overestimation among parents serve to experience their children as less skewed than in reality for their gender and age, thereby challenging a realistic view of the weight status of the offspring.

Parents’ weight misperception seems to affect how worried they are about the weight status of their children [24]. More specifically, parents who underestimate the weight status of overweight children are less concerned with their weight than those who recognize their children as overweight [25]. It has also been reported that parents of obese children, who do not recognize weight problems in their offspring, are less likely to let their child be a part of an intervention program for obesity [26]. In line with this, it has been suggested that parents with an inaccurate weight perception also report less concerns about their children’s lifestyle in terms of eating habits, physical activity and fitness level [18]. However, to our knowledge, no previous studies have investigated how parental weight perception relates to evaluation of more general emotional and behavioral problems, in addition to disturbed eating patterns. There is also a need to investigate the relation between parent- and self-reported weight perceptions in order to get a better understanding of the informant effects involved. It has been reported that weight perception accuracy among children and parents is significantly related [27]. Another study found that although the parents were slightly more accurate than their teens, both informants were poor at recognizing obesity [28]. Yet, this study focused solely on the perception of excessive weight, and it would be interesting to investigate the relation between self- and parent-report with a broader range of weight categories in a sample of young adolescents.

### Aims and hypotheses

This study aimed to explore the weight perception accuracy among parents of young adolescents. The purpose was three-fold:

1) To assess the distribution of parental over- and underestimation in contrast to a more accurate perception, the latter defined as a ‘control condition’, across gender and weight status.

2) To investigate whether the parent’s weight perception accuracy was related to their reports on their offspring’s disturbed eating patterns and mental health problems.

3) To identify risk factors for parental over- and underestimation across gender, including weight status, the adolescents’ own weight perception accuracy and parent reports on disturbed eating patterns as possible predictors.

All data were based on parent reports besides weight perception accuracy, which was also assessed for the adolescents. Following the notion of a positivity bias among parents, we expected highest perceptual inaccuracies for adolescents with under- or overweight. We also hypothesized that weight misperception would be related to the parents’ reports on disturbed eating patterns and mental health problems of the adolescents, with lower symptom scores for those with under- or overweight who were misperceived as being of normal weight. Finally, we assumed that over- and underestimation among parents would be predicted by BMI, their concerns about eating patterns and mental health problems as well the adolescents’ own weight perception accuracy.

## Methods

### Participants

The study is part of the longitudinal Bergen Child Study (BCS) in Norway, where a cohort of children born between 1993 and 1995 from all public and private schools in the municipality of Bergen were followed up over a ten-year period. Among the aims of the BCS is to estimate prevalence data for a broad specter of mental health problems, and use of health and school services. The ethics of the study as a whole and the different waves of the project were approved by the Regional Committee for Medical Research in Western Norway. The procedure of the BCS is described in more detail elsewhere [29].

The present analyses are drawn from the first phase of the second wave of the study administered during spring 2006. A four-page screening questionnaire was handed out from the schools to the children as well as their parents and teachers, and written informed consent was attained from all parents whose children were included in the study. The sample consisted of 5,781 subjects aged 11–13 years equally divided between 5^th^, 6^th^ and 7^th^ grade in elementary school. There were slightly more girls (52.5%) than boys. The questionnaires were usually completed by the mother (63.9%) or by both parents (17.8%).

The number of participants reflected a participation rate from the total population of 61%. Previous reports on the representativeness of the study have concluded that there was a significant non-response bias with respect to the measures on emotional and behavioral problems in the first wave of the BCS [30], although this difference was small in magnitude.

### Measures

#### Demographic variables

The participants were asked to mark their gender (girl or boy), school grade (5–7), and pubertal timing. The latter was assessed by the statement “How would you describe your pubertal development” which could be rated on a Likert scale from 1 (*a lot earlier than my peers*) to 5 (*a lot later than my peers*). SES was based individual indicators rather than an aggregated measure [31] and included parent-reported ratings of family economy from 1 (*very poor*) to 5 (*very good*) and parental education from elementary school (1) to university level (5).

#### Weight status

Body Mass Index (BMI) was calculated as weight/height^2^ based on parents’ report of their children’s weight and height. The continuous BMI measure was recoded to three weight categories (underweight, normal weight and overweight) based on international guidelines for age- and gender specific BMI percentiles [32]. Accordingly, BMI of less than 5^th^ percentile was classified as underweight, from 5^th^ to less than 85^th^ as normal weight, from 85^th^ to less than 95^th^ percentile as overweight, and more than 95^th^ percentile as obesity. Obese subjects were included in the overweight group.

#### Parents’ weight perception

The parents’ score on “How would you evaluate the child’s weight?” on a scale from 1 (*too thin*) to 5 (*too fat*) was contrasted with the children’s BMI status to define three weight perception groups: overestimation, accurate and underestimation. Adolescents with underweight could be either overestimated or correctly perceived, while adolescents with normal weight could also be underestimated. Overweight adolescents could be either underestimated or correctly perceived. An accurate weight perception defined a control condition.

#### Adolescents’ weight perception

The adolescent’s score on the statement “I feel too fat” on a scale from 1 (*not true*) to 4 (*certainly true*) was contrasted with parent reported BMI and classified in three weight perception groups. Subjects with actual under- or normal weight and feeling too fat were placed in the overestimation group, while subjects with actual overweight and not feeling overweight defined the underestimation group. Only the extreme scores were used in the categorization to avoid misinterpretations of the results. This implied that subjects with under- and normal weight who rated the question of feeling too fat as “certainly true” were included in the overestimation group, while those rating this question more moderately as “true” were included in the control condition. Likewise, only overweight subjects who rated this question as “not true” were included in the underestimation group. Participants with an accurate weight perception were included as controls for each weight category.

#### Disturbed eating patters

Eating patterns were assessed by four adjusted items from the Eating Disorders Scale (EDS-5) and six supplementary items regarding eating attitudes and behaviours (see Tables 1 and 2). EDS-5 was developed by Rosenvinge and colleagues for screening use [33] and has in its complete form been shown to be sensitive (0.90) and specific (0.89) in detecting symptoms of eating disorders when compared to DSM-IV criteria. The scale usually collects self-report data, but was adjusted to parent reports in this study. The items included from EDS-5 addressed comfort eating, feeling too fat, feeling guilty after eating, and needing strict diets to feel in control of eating among the adolescents. Supplementary items covered whether the adolescents have deliberately lost weight, have disturbed thoughts about weight or shape, are a vegetarian and/or have chosen a diet different from the rest of the family. All items were rated on a 3-point Likert scale from *not true* to *certainly true.*

**Table 1:** **Clinical characteristics of girls with underweight, normal weight, and overweight by weight perception accuracy**^**n **^**presented as means (SD)**

**Weight perception accuracy**	**Underweight**		**Normal weight**			**Overweight**	
	**Accurate**	**Overestimation**	**Underestimation**	**Accurate**	**Overestimation**	**Accurate**	**Underestimation**
Background variables							
Grade	1.91 (0.83)	1.97 (0.83)	1.87 (0.81)	1.98 (0.82)	1.86 (0.81)	1.70 (0.77)	1.80 (0.68)
Pubertal timing	3.51 (0.71)	3.31 (0.82)	3.15 (0.73)**	2.93 (0.72)	2.85 (0.63)	2.67 (0.83)	2.64 (0.84)
Family economy	2.14 (0.67)	2.15 (0.73)	2.22 (0.79)	2.15 (0.74)	2.19 (0.75)	2.26 (0.73)	2.45 (0.84)
Parental education	3.38 (1.16)	3.46 (1.15)	3.45 (1.04)	3.48 (1.11)	3.21 (1.13)*	3.20 (1.09)	2.93 (1.11)
Disturbed eating patterns							
*Consciously lost weight*	0.03 (1.62)	0.00 (0.00)	0.07 (0.29)*	0.03 (0.19)	0.10 (0.30)**	0.16 (0.44)	0.11 (0.31)
*Disturbed thoughts about weight/shape*	0.09 (0.34)	0.02 (0.14)*	0.13 (0.36)***	0.04 (0.22)	0.14 (0.41)***	0.17 (0.45)	0.11 (0.35)
*Shows comfort eating*	0.01 (0.12)	0.03 (0.17)	0.03 (0.23)	0.02 (0.14)	0.18 (0.47)***	0.38 (0.64)	0.07 (0.25)***
*Feels guilty after eating*	0.01 (0.1)	0.01 (0.10)	0.06 (0.27)*	0.02 (0.16)	0.17 (0.44)***	0.26 (0.53)	0.07 (0.25)**
*Follows strict diets/rituals*	0.01 (0.12)	0.01 (0.10)	0.04 (0.20)***	0.00 (0.07)	0.04 (0.21)***	0.06 (0.26)	0.00 (0.00)
*Is a vegetarian*	0.04 (0.20)	0.04 (0.24)	0.05 (0.29)	0.02 (0.17)	0.00 (0.00)	0.02 (0.19)	0.00 (0.00)
*Provokes vomiting*	0.00 (0.00)	0.02 (0.14)	0.00 (0.00)	0.00 (0.04)	0.00 (0.00)	0.01 (0.17)	0.00 (0.00)
*Is happy about physical appearance*^	0.47 (0.58)	0.39 (0.53)	0.54 (0.62)	0.45 (0.62)	0.80 (0.66)***	0.88 (0.60)	0.57 (0.58)***
*Says he/she feels too fat*	0.11 (0.39)	0.07 (0.26)	0.08 (0.30)**	0.20 (0.45)	0.73 (0.73)***	0.99 (0.77)	0.63 (0.63)***
*Has chosen own diet*	0.05 (0.23)	0.00 (0.00)*	0.06 (0.24)**	0.02 (0.16)	0.05 (0.27)*	0.05 (0.25)	0.01 (0.12)
Mental health problems							
Emotional problems	1.60 (2.11)	1.22 (1.56)	2.03 (2.09)***	1.09 (1.58)	1.65 (1.75)**	2.03 (2.14)	1.67 (1.83)
Conduct problems	0.79 (1.03)	0.76 (1.00)	0.88 (1.56)*	0.66 (0.97)	0.99 (1.26)**	1.15 (1.33)	0.83 (1.31)
Hyperactivity/inattention	2.03 (1.85)	1.97 (1.69)	2.18 (1.90)*	1.82 (1.73)	2.00 (1.83)	2.28 (1.96)	2.45 (1.04)
Peer problems	1.11 (1.76)	1.09 (1.66)	1.42 (1.85)***	0.74 (1.31)	1.39 (1.89)	1.71 (2.00)	1.09 (1.51)*

^n^*Note*. Group differences relative to correspondence significant at *p < .05, **p < .01 and ***p < .001.

^Scores reversed.

**Table 2:** **Clinical characteristics of boys with underweight, normal weight, and overweight by weight perception accuracy**^**n **^**presented as means (SD)**

**Weight perception accuracy**	**Underweight**		**Normal weight**			**Overweight**	
	**Accurate**	**Overestimation**	**Underestimation**	**Accurate**	**Overestimation**	**Accurate**	**Underestimation**
Background variables							
Grade	1.93 (0.81)	1.85 (0.86)	2.04 (0.83)	1.98 (0.81)	1.99 (0.79)	1.82 (0.79)	1.88 (0.64)
Pubertal timing	3.15 (0.68)	3.02 (0.67)	2.97 (0.68)	2.91 (0.59)	2.95 (0.79)	2.80 (0.89)	2.50 (0.71)
Family economy	2.19 (0.74)	2.08 (0.80)	2.26 (0.80)	2.16 (0.72)	2.35 (0.80)**	2.45 (0.86)	2.30 (0.82)
Parental education	3.33 (1.20)	3.47 (1.10)	3.27 (1.11)	3.45 (1.10)	3.07 (1.16)***	2.98 (1.06)	3.21 (1.29)
Disturbed eating patterns							
*Consciously lost weight*	0.02 (0.13)	0.00 (0.00)	0.06 (.28)	0.03 (0.19)	0.11 (0.33)***	0.19 (0.50)	0.19 (0.50)
*Disturbed thoughts about weight/shape*	0.08 (0.28)	0.00 (0.00)*	0.06 (0.23)*	0.02 (0.17)	0.09 (0.30)***	0.21 (0.48)	0.40 (0.52)
*Shows comfort eating*	0.00 (0.00)	0.00 (0.00)	0.03 (0.19)	0.02 (0.15)	0.19 (0.43)***	0.38 (0.60)	0.20 (0.42)
*Feels guilty after eating*	0.07 (0.31)	0.00 (0.00)	0.04 (0.19)*	0.01 (0.12)	0.13 (0.35)***	0.22 (0.46)	0.20 (0.42)
*Follows strict diets/rituals*	0.03 (0.26)	0.00 (0.00)	0.00 (0.00)	0.01 (0.11)	0.04 (0.23)**	0.10 (0.31)	0.10 (0.32)
*Is a vegetarian*	0.00 (0.00)	0.02 (0.13)	0.04 (0.25)***	0.00 (0.06)	0.00 (0.00)	0.00 (0.00)	0.10 (0.32)**
*Provokes vomiting*	0.00 (0.00)	0.00 (0.00)	0.01 (0.16)	0.00 (0.06)	0.02 (0.18)*	0.04 (0.21)	0.10 (0.32)
*Is happy about physical appearance*^	0.47 (0.60)	0.27 (0.60)	0.32 (0.54)	0.30 (0.56)	0.71 (0.67)***	0.90 (0.56)	0.60 (0.52)
*Says he/she feels too fat*	0.05 (0.22)	0.05 (0.28)	0.04 (0.23)	0.08 (0.31)	0.65 (0.70)***	1.12 (0.77)	0.60 (0.84)
*Has chosen own diet*	0.07 (0.25)	0.02 (0.13)	0.08 (0.34)***	0.02 (0.15)	0.06 (0.26)**	0.04 (0.21)	0.10 (0.32)
Mental health problems							
Emotional problems	2.08 (2.09)	0.78 (1.35)***	1.43 (1.81)***	0.93 (1.40)	1.86 (2.22)***	1.62 (2.03)	3.20 (2.94)*
Conduct problems	1.41 (1.78)	0.62 (0.89)**	1.02 (1.34)**	0.77 (1.12)	1.45 (1.75)***	1.09 (1.47)	1.10 (1.66)
Hyperactivity/inattention	4.15 (2.89)	2.32 (1.93)***	2.82 /2.51)*	2.46 (2.09)	3.33 (2.52)***	3.10 (2.19)	4.00 (2.94)
Peer problems	1.76 (2.28)	0.65 (1.08)**	1.29 (1.71)*	0.97 (1.55)	1.93 (2.35)***	2.41 (2.05)	2.50 (2.17)

^n^*Note*. Group differences relative to correspondence significant at *p < .05, **p < .01 and ***p < .001.

^Scores reversed.

#### Mental health problems

General mental health, defined by emotional and behavioral problems, was assessed by the parent reports on the symptom scales in the Strength and Difficulties Questionnaire (SDQ). This widely applied questionnaire was developed by Goodman for use in non-clinical samples [34,35]. Each scale consists of five items formulated as statements that the subjects rate on a 3-point Likert scale from *not true* to *certainly true*. The scales have been validated in several epidemiological studies and found to have good psychometric qualities, also in the Bergen Child Study [29] as well as other comparable Norwegian samples [36].

The four symptom scales included in the present study are denoted a) Emotional problems (SDQ-Emo), b) Conduct problems (SDQ-Con), c) Hyperactivity-Inattention problems (DQ-Hyp), and d) Peer problems (SDQ-Peer). The total problem score range from 0 to 10 for all four scales.

### Design and statistical analyses

Statistical analyses were performed with SPSS package, version 17.0. Distribution of categorical variables was presented as percentages and continuous variables as mean with standard deviations. All analyses were done separately for girls and boys, and missing data was replaced by the item’s mean score for subjects of same gender. Preliminary correlation analyses were run to test concordance between parental weight evaluation and related variables for girls and boys, using Pearson’s two-tailed tests. The correspondence between the weight perception among parents and adolescents was also investigated by correlation analyses. We assessed both the concordance for weight perception per se split by gender, and the weight perception accuracy among the informants split by gender and weight status.

The weight perception groups were compared on demographic and clinical variables split by gender and weight status by independent samples *t* tests. Logistic regression analyses were performed to identify variables related to the risk of over- and underestimation split by gender. These analyses were organized in three blocks; block 1 included the demographic variables age, pubertal development and SES, block 2 included weight status and the adolescents’ own weight perception accuracy, and block 3 included parent-reported eating patterns. In block 2, ‘normal weight’ and ‘control’ defined the reference categories for weight status and weight perception, respectively. Goodness-of-fit was tested for each block of the analyses using the Hosmer-Lemeshow test. The estimates in the regressions analyses were reported as Odds Ratio (OR) with 95% Confidence Interval (CI), and the total explained variance of each model (*R*^2^) was calculated with Nagelkerke R Square.

## Results

### Descriptive statistics

#### Sample characteristics

With regard to BMI status, 7.8% of the girls were classified as underweight, 82.7% as normal weight and 9.5% as overweight. For boys, the distribution was 6.0% underweight, 90.3% normal weight, and 3.7% overweight.

The participants formed an ethnically homogenous and socioeconomically advantaged sample. The majority of the parents were born in Norway (> 85%), and more than 40% of the parents reported education on university level. Furthermore, 68% of the parents rated their family income as good, 29% as moderate, and 3% as poor. The adolescents’ health was generally described as good by the parents (> 95%).

#### Weight perception accuracy

Regardless of gender and BMI, 81.75% of the parents showed an accurate weight perception while 9.8% overestimated and 8.45% underestimated the weight status of their offspring. The weight perception accuracy among the parents varied by gender and weight status (see Table 3). The parents overestimated more than half of the underweight girls and boys. Among normal weight subjects, less than 10% of the girls and boys were estimated as either over- or underweight by their parents. More than one third of the parents of overweight girls underestimated the weight of their child, while this was the case for only around 12% of the boys.

**Table 3:** Parental weight perception accuracy (%) by weight status among girls and boys

	** Weight status**	***n***	**Weight perception accuracy**		
			**Underestimation**	**Accurate**	**Overestimation**
**Girls**	Underweight	237	0.0	42.4	57.6
	Normal weight	2509	6.2	88.9	4.9
	Overweight/obese	289	34.2	65.8	0.0
**Boys**	Underweight	165	0.0	48.4	59.6
	Normal weight	2480	8.8	82.3	8.9
	Overweight/obese	101	12.8	87.2	0.0

The adolescents’ own weight perception accuracy also varied by gender and weight status. A total of 5.9% of the girls and 3.7% of the boys with under- or normal weight overestimated their own body size, while 19.3% of the girls and 20.5% of the boys with overweight showed a pattern of underestimation.

The correspondence between weight perception per se among parents and adolescents was significantly associated for both genders, with a correlation between the parental weight perception and the adolescents’ perception of being overweight of *r* = .368 for girls and *r* = .394 for boys, with *p* < .01 for both measures. The parents’ and adolescents’ weight perception accuracy regardless of weight status was only significantly associated for girls (*r* = .088, *p* < .01). However, performing the analyses split by weight status revealed that the weight perception accuracy among the informants was significantly associated for both overweight girls (*r* = .257, *p* < .01) and boys (r = .242, *p* < .05).

### Parental weight perception, demographic factors and clinical variables

The weight perception groups were compared on demographic and clinical variables split by actual weight status, and the results of these comparative analyses are presented separately for girls and boys in Tables 1 and 2.

#### Underweight adolescents

Comparisons between underweight adolescents who were overestimated and accurately perceived by their parents are presented in Table 1 for girls and Table 2 for boys. All reported comparisons were significant at *p* < .05. Girls with underweight who were overestimated girls were reported to choose their own diet less frequently than those who were accurately perceived, while overestimated boys were reported to have less mental health problems than the accurate group. For both genders, parents who overestimated also reported less disturbed thoughts about food/weight on behalf of their offspring.

#### Normal weight adolescents

Comparisons between normal weighted adolescents who were underestimated, accurately perceived, and overestimated by their parents are presented in Table 1 for girls and Table 2 for boys. Girls of normal weight who were underestimated were also reported to have later pubertal development, higher levels of feeling guilty after eating, following strict diets/rituals, and choosing their own diet, but lower scores on feeling too fat, relative to the accurate group. Boys with normal weight who were underestimated were viewed as having more disturbed eating patterns on 4 of 10 items when compared to the accurate group. For both genders, underestimation was associated with significantly higher mental health problems than the accurate group.

For girls with normal weight, overestimation was associated with lower parental education level and higher scores on disturbed eating patterns on 8 out of 10 items. For boys with normal weight, overestimation was associated with better family income, lower parental education as well as higher scores on disturbed eating patterns on 9 out of 10 items relative to the accurate group. For both genders, overestimation was associated with significantly higher scores on all mental health problems based on the parent-reports.

#### Overweight adolescents

Comparisons between overweight adolescents who were overestimated and accurately perceived by their parents are presented in Table 1 for girls and Table 2 for boys. These comparisons revealed few significant differences. However, overweight girls who were underestimated were also scored lower on comfort eating, feeling guilty after eating, not being happy with physical appearance, feeling too fat, and having peer problems than the controls. Overweight boys who were underestimated more often tended to be vegetarian and showed higher emotional problems than the control group according to the parents’ reports.

### Predictors of parental weight misperception

#### Variables related to overestimation

Variables related to the risk of overestimation among the parents varied partly by gender. After controlling for demographic variables, parents of both genders were more likely to overestimate if their offspring was underweight (girls: OR 16.64, CI 11.18 - 24.77, *p* < .001, boys: OR 5.85, CI 2.92 – 11.71, *p* < .001), and if the adolescents themselves overestimated their weight status (girls: OR 3.47, CI 1.60 – 7.51, *p* < .01, boys: OR 3.39, CI 1.11 – 10.37, *p* < .05). In addition, some aspects of disturbed eating patterns were related to the risk of overestimation; parent reports on comfort eating in both genders (girls: OR 4.42, CI 1.96 - 10.01, *p* < .001), boys: OR 3.04, CI 1.10 – 8.43, *p* < .05) as well as feeling too fat among boys (OR 1.65, CI 1.01 – 2.69, *p* < .05). The regression models explained considerably more variance for boys (*R*^2^ = .702) than for girls (*R*^2^ = .275).

#### Variables related to underestimation

For parents of underweight girls, the risk of underestimation increased with overweight (OR 6.63, CI 3.84 – 11.43, *p* < .001), underestimation among the adolescents themselves (OR 2.54, CI 1.07 – 6.03, *p* < .05), and parental reports on disturbed thoughts about food/weight (OR 3.31, CI 1.80 – 6.08, *p* < .001). Underestimation on behalf of girls was also negatively associated with underweight (OR 0.13, CI 0.02 – 0.99, *p* < .05) as well as parental reports on comfort eating (OR 0.29, CI 0.13 – 0.65, *p* < .001) and feeling too fat (OR 0.46, CI 0.31 – 0.70, *p* < .001).

Among the boys, neither the weight status nor the adolescents’ own weight perception accuracy were significant predictors. Only a low level of feeling too fat (OR 0.32, CI 0.14 – 0.72, *p* < .01) as reported by the parents was related to the risk of underestimation after controlling for demographic factors. However, other disturbed eating patterns approached significance, for instance if the offspring has consciously lost weight (OR 2.17, CI 0.97 – 4.88, *p* = .060) or showed disturbed thoughts about food/weight (OR 2.70, CI 0.98 – 7.41, *p* = .054) according to the parents. The total models for underestimation explained more variance for girls (*R*^2^ = .142) than for boys (*R*^2^ = .080).

## Discussion

This study aimed to add to the knowledge on weight perception by addressing the accuracy of parent reports in relation to a broad range of demographic and clinical variables. There were three main findings. Firstly, a substantial proportion of the parents misperceived the weight status of adolescents classified as under- or overweight, correcting for deviations from the average as a possible positivity bias. Secondly, parents who accurately perceived their offspring as under- or overweight were most concerned as regarded disturbed eating patterns and mental health problems on behalf of the adolescents. Third, after controlling for demographic factors, significant predictors of over- and under-estimation by the parents were the weight status of the offspring, the adolescents’ own weight perception accuracy, as well as some disturbed eating patterns. Together, the multiple findings of this study support a positivity bias among parents, especially with regard to offspring classified with under- or overweight, and emphasize the reciprocal influence between the weight perception among the adolescents and their parents.

The finding that over half of the *underweight* adolescents were overestimated by the parents is compatible with findings from previous studies [18]. However, it has also been reported that up to 70% of parents of underweight children tend to overestimate their weight status [19]. Age differences in the offspring can be an explanation for these varied findings, as parental weight misperception generally seems to be higher for younger children [12]. A positivity bias among the parents might explain this pattern of overestimating on behalf of offspring with underweight, and thereby minimizing the deviation from normal weight. In addition, the masking effect of clothes could make it especially difficult for parents to estimate the size of underweight children [19] and thereby explain the relatively high frequency of this misperception.

Parents of *normal* weight adolescents either over- or underestimated their weight status in about one fifth of the cases. This proportion is moderate when compared to the over- and underestimation that appeared in the other weight categories. It may demonstrate that parents generally struggle with estimating the weight status of their adolescents, but that inaccuracies increase when the actual weight status is below or above average. This is also in accordance with a possible positivity bias, as there is less likelihood of distorting the weight of normal weight children.

With regard to gender differences, parents more frequently overestimated boys (8.9%) than girls (4.9%) of normal weight. As the body ideal for males is currently muscular rather than slim, this could be another expression of a skewed perception among parents adjusting the view of their offspring towards positive and attractive features for their gender.

Among *overweight* adolescents, parents underestimated 34.8% of the girls and 12.8% of the boys, respectively. Although this shows a substantial misperception, underestimation of overweight has been reported to be even more common for younger overweight children [37]. Parental underestimation was more common for girls than boys in the present sample, and other authors have reported the same gender difference [16,38]. If a positivity bias is driving the perceptual inaccuracy, it is reasonable that the underestimation of overweight is especially salient for girls due to the dominant ideal of being slim for females and the stigma related to being overweight.

The reports on eating patterns and mental health problems varied considerably across the actual weight categories as well as the accuracy of the parents’ weight perception. For the underweight adolescents, the most consistent differences emerged on mental health problems among boys, as parents who overestimated the weight status of their sons also described significantly lower symptom scores. Normal weight adolescents who were overestimated presented more disturbed eating patterns than those who were underestimated when compared to the controls, but both misperceptions were related to elevated scores on mental health problems based on the parent reports.

For overweight adolescents, there were few significant differences between those who were underestimated and the controls, apart from lower scores on some aspects of disturbed eating patterns and peer problems among the girls. Overweight boys who were underestimated also presented more emotional problems than the controls, according to the parents. Seen together, the results suggest a pattern in which those parents who perceived their offspring to be closer to normal weight than their BMI suggests, also believed they had less disturbed eating patterns and mental health problems than the controls. Mathieu and colleagues [18] reported a comparable trend where the description of the lifestyle profile of children depended systematically on the parents’ weight perception accuracy. One possible interpretation of this might be that positivity biases influencing the parents’ weight estimates also skew the beliefs about the psychosocial well-being of the adolescents in an optimistic way. However, the results for the normal weight adolescents were less consistent in the present sample, and this supports the idea that the biases in parental perception are most visible with actual deviation from normal weight. In addition, the emotional problems reported on behalf of overweight boys who were underestimated suggest that parents are able to recognize some problems among their offspring even if they are not aware of their specific weight problems.

Overall, the regression analyses supported the hypotheses that the parents’ weight misperceptions varied systematically with the weight status of the offspring. Accordingly, the risk of overestimation was significantly predicted by being underweight, which replicates the parents’ tendency to minimize deviations from average weight [20]. In addition, the adolescents’ own perception accuracy proved a significant predictor, as underestimation among the offspring increased the odds by threefold for the same misperception among parents. This concordance is interesting for the understanding of the sociocultural mechanisms involved in weight misperception and highlights the reciprocal influence between parents and children regarding distortions in body perception. Comfort eating also affected overestimation significantly for both genders, increasing the odds of this misperception by more than threefold. To our knowledge, the relation between parental overestimation and reports on comfort eating among adolescents has not previously been reported. Moreover, boys whose parents believed that they felt too fat showed a higher likelihood of being overestimated. These findings may add to knowledge of distinct eating patterns and concerns among adolescents that are related to overestimation among parents besides body weight.

In sum, the regression models for overestimation were best able to predict misperception on behalf of the boys, with over 70% of the variance explained for boys relative to less than 30% for girls. This may support the notion that body image among boys is influenced by more distinct variables than girls [39], and that the same mechanisms might come into play with regard to parents’ estimates of the weight status of adolescents. The diverse results in the present study suggest that there is need for more knowledge on factors relevant to parental overestimation, both with regard to prevalence, gender differences as well as consequences for the parents’ concerns about the mental health of their children and engagement in healthy weight control.

The regression models for underestimation revealed more gender differences than for overestimation with regard to significant predictors. Weight status defined as overweight and self-reported underestimation only predicted parental underestimation for girls in the sample. In addition, underestimation among girls was significantly related to high scores on disturbed thoughts about food/weight as well as low scores on comfort eating and feeling too fat. In contrast, neither the weight status nor the adolescents’ own weight perception accuracy significantly affected underestimation among boys.

After controlling for demographic factors, only low scores on feeling too fat were related to the risk of underestimation among parents of adolescent boys. This factor proved to be significant for both genders, implying that parents who reported little concern about being too fat among their offspring also tended to underestimate their weight. As noted earlier, the opposite association was reported for overestimation among boys, as high scores on feeling too fat increased the likelihood of this misperception. These findings add support to the idea of a reciprocal influence between parents and their children with regard to body image and weight misperceptions. This highlights that eating patterns and attitudes conveyed by adolescents may affect the way the parents’ view of their weight status, in addition to the biases that tend to minimize deviation from average.

The regression models were best able to predict underestimation among girls, with an explained variance of about 14% relative to 8% for boys. This might be due to the generally higher frequency of parental underestimation for girls reported in other samples [16]. Yet, the relatively low explained variance for both genders suggest that there are variables besides the ones included in the present study that contribute to parents’ tendency to minimize the weight status of their children.

### Strengths and limitations

The study has limitations that should be noted. First, as a cross-sectional design, the results are not open for causal interpretations. Second, BMI was based on parent-reported weight and height instead of direct measures due to the large sample size and practical challenges with individual assessments. One could argue that the parents were biased in both their report on specific weight and height in addition to the perception of the adolescents’ weight status, but the results showed substantial discrepancies between the weight and height that was reported and the perception of the BMI status of the adolescents. If the reports on actual BMI were consistently biased, we would most likely not have found the patterns of over- and underestimation that is comparable with previous studies on parental weight perception. One could therefore argue that the results are even more striking for the parents who misperceived the weight status of their offspring despite having reported a height and weight that suggested deviations from the average.

A third limitation is the lack of a complete measure for the parents’ concerns about body image and eating concerns on behalf of the adolescents. We used ten items, of which four were adjusted from the EDS-5. Ideally, this scale should have been used in its complete form and included in the regression analyses as a sum score, as well as being compared with the reports from the adolescents. On the other hand, single questionnaire items have been used in comparable studies in order to get a more detailed understanding of factors related to parental weight misperception [37,38]. The items that were chosen in the present study also seemed to be specifically related to over- and underestimation across the different weight categories, and this gives valuable insights for preventive and clinical work. Yet, although the EDS-5 is one of the few screening instruments for eating disturbances developed in Norway [40], it has so far only been validated in samples with 18 year olds and above [33]. Further studies needs to be done in order to establish norms for younger samples as well.

A further possible problem is that the study employed different measures for weight perception accuracy, as weight perception was assessed by a rating of weight status among the parents and by feeling overweight among the adolescents, respectively. Furthermore, which of the parents who completed the interview was not included in the analyses, and it is possible that the results were influenced by the dominance of mothers’ reports in the study.

Possible strengths include that, to our knowledge, this is the first study of parental over- and underestimation across different weight categories for girls and boys in relation to disturbed eating patterns, emotional problems, and behavioral problems. With data from an ongoing epidemiological study with a large and representative sample, we could draw well-grounded conclusions regarding weight misperception among the parents and its’ association with concerns about psychosocial problems among their offspring. The adolescent’s own weight perception accuracy was also included in order to evaluate the concordance between the two informants.

### Implications for future research and preventive interventions

Research on weight perception accuracy among adolescents and their parents is relatively new, and knowledge in this field is important in the face of the common eating and weight concerns in modern culture. Future research on parental weight misperceptions should ideally be longitudinal to allow for causal explanations for the different misperceptions and to formulate a more thorough theoretical understanding of the psychological mechanisms involved. It is also essential to include all weight categories as both the distribution and factors that influence the inaccuracy seem to vary according to the BMI of the children. Although most studies have focused on underestimation of excessive weight, overestimation of under- and normal weight should be further investigated in order to get a more comprehensive picture of the body perception among youth and their parents.

The notion of a positivity bias was to a large part supported by the present study, but more research is needed to establish how these biases operate and to which extent they modify the experience of the psychosocial health of the offspring in addition to physical features as weight status. In this sample, there was a pattern of lower symptom scores for adolescents who were erroneously perceived as normal weight, and this suggest that parents who minimize weight problems are also inclined to report less eating concerns and mental health problems in their offspring. Positivity biases can have an adaptive function by preserving a stable and optimistic impression of self and others, but if they also lower parents’ awareness of real problems in their offspring, the skewedness in perception could challenge parent’s ability to meet the needs of their children and thereby impair healthy development. Future studies on parental weight estimates might also take account of the degree of perceptual inaccuracy in order to define nuanced subgroups in the samples and to provide reference points for the clinical significance of the various misperceptions.

Based on these multiple findings, preventive and clinical interventions should target weight perception among not only children and adolescents, but also their parents. This perspective is essential for supporting parents’ ability to estimate the weight status of their offspring in a realistic way, and subsequently, seek help early and initiate appropriate interventions if needed. The high frequency of weight misperception of under- and overweight adolescents suggests that interventions should be directed towards the whole family, including education on weight perception accuracy [15] as well as supporting health literacy in general [41]. Strategies for improving parental weight perception has been shown to be successful [42], and parents tend to be more worried about the weight status of their child if their doctor express similar concerns [13]. This gives hope for interventions directed at improving the parents’ awareness of weight deviations in their children and offering a counterbalance to the positivity bias that might skew the parents’ perceptions of the physical and emotional well-being of their offspring.

## Conclusion

The findings suggest that parents misperceive the weight status of young adolescents in line with the adolescents themselves. Parents were inclined to overestimate the weight status of underweight adolescents and underestimate the weight status of overweight adolescents. The low awareness of deviations from normal weight among parents may challenge healthy weight maintenance efforts as well as responsiveness to psychosocial problems on behalf of adolescents. Family-oriented interventions should target potential positivity biases and work towards a realistic perception of self and others in order to encourage healthy weight control and adequate emotional support.

## Competing interests

The authors declare that they have no competing interests.

## Authors’ contributions

LS participated in the design of the study, performed the statistical analyses and was responsible for drafting the manuscript. BL participated in the formulation of the aims of the study, interpretation of the main findings and drafting the manuscript. MH participated in the planning of statistical analyses and commented on written drafts. KMS participated in the design and coordination of the study and helped to formulate the research questions and draft the manuscript. All authors read and approved of the final manuscript.
